# Genome-Wide Identification of *PRP* Genes in Apple Genome and the Role of *MdPRP6* in Response to Heat Stress

**DOI:** 10.3390/ijms22115942

**Published:** 2021-05-31

**Authors:** Xiaoli Zhang, Xiaoqing Gong, Danyang Li, Hong Yue, Ying Qin, Zhu Liu, Mingjun Li, Fengwang Ma

**Affiliations:** State Key Laboratory of Crop Stress Biology for Arid Areas/Shaanxi Key Laboratory of Apple, College of Horticulture, Northwest A & F University, Yangling 712100, China; xlzhang0725@nwafu.edu.cn (X.Z.); gongxq0103@nwsuaf.edu.cn (X.G.); ldanyang@nwafu.edu.cn (D.L.); yyhong19972020@163.com (H.Y.); qinying883@163.com (Y.Q.); lz2020jy@163.com (Z.L.)

**Keywords:** proline-rich protein, apple genome, expression profiles, heat stress, ROS

## Abstract

Plant proline-rich proteins (PRPs) are cell wall proteins that occur in the plant kingdom and are involved in plant development and stress response. In this study, 9 *PRP* genes were identified from the apple genome and a comprehensive analysis of the *PRP* family was conducted, including gene structures, phylogenetic analysis, chromosome mapping, and so on. The expression of *MdPRPs* varied among tissues and in response to different types of stresses. *MdPRP4* and *MdPRP7* were induced by five detected stress treatments, including heat, drought, abscisic acid, cold, and salt; the expression patterns of the others varied under different types of stress. Subcellular localization showed that MdPRPs mainly functioned in the cytoplasm, except for MdPRP1 and MdPRP5, which also functioned in the nucleus. When *MdPRP6* was overexpressed in tobacco, the transgenic plants showed higher tolerance to high temperature (48 °C) compared with wild-type (WT) plants. The transgenic plants showed milder wilting, a lower accumulation of electrolyte leakage, MDA and ROS, and a higher level of chlorophyll and SOD and POD activity, indicating that *MdPRP6* may be an important gene in apples for heat stress tolerance. Overall, this study suggested that MdPRPs are critically important for the ability of apple responses to stresses.

## 1. Introduction

Sessile plants are continually challenged by various biotic and abiotic stresses throughout their life cycles which not only affect their distribution, but also threaten crop yields. Abiotic stresses such as high temperature, drought, and salt damage are the most prominent and widespread. Global surface temperature is expected to increase by 2–4 °C by the end of the 21st century [1]. Extreme heat is harmful to plants, as high temperature stress leads to protein denaturation and aggregation, the loss of plasma membrane integrity, and the accumulation of reactive oxygen species (ROS), which disrupts cell metabolism and homeostasis. Under high temperature conditions, the fluidity of the lipid bilayer structure of plant cell membranes is significantly enhanced, which can cause electrolyte leakage, reactive oxygen generation, and oxidative damage. Plants accumulate antioxidants, osmoprotectants, and other metabolites to cope with heat stress damage through different pathways [2]. The protective enzymes and antioxidants in chloroplasts and mitochondria are also important for reducing oxidative damage [3].

Plant proline-enriched proteins (PRPs) contain proline and hydroxyproline and are widely distributed in plants. The most notable feature of PRPs is their high content of proline (Pro) residues, which often include at least two consecutive Pro arranged in the amino acid chain. Plant PRPs can be divided into three categories according to the presence or absence of N-terminal signal peptides and differences in domains: (I) PRPs with two domains containing N-terminal signal peptides and rich Pro repeat regions; (II) PRPs with three domains containing an N-terminal signal peptide, Pro-rich repeat region, and C-terminal cysteine-rich region, and (III) PRPs with no signal peptide at the N-terminal and several similar PPVYK repeat sequences at the C-terminal. Type II PRPs can be divided into two subtypes according to the quantity and distribution of cysteine. There are four or six cysteine residues at the C-terminal in a fixed pattern (...C…C…C…C…C…C…) or eight cysteines arranged in a specific manner (…C…C…CC…CXC…C…C…) [4]. The presence of signal peptides indicates that PRPs are likely secreted [5,6,7]. At the same time, because of the presence of terminal hydrophobicity, PRPs can also be classified into other protein families, such as the 8 CM (eight-cysteine motif) domain protein or LTP (lipid transfer protein) [8,9].

The expression of *PRP* genes is regulated by the growth and development of plants, which can show organ specificity, tissue specificity, and even cell specificity. For example, *PdPRP* in poplars is mainly expressed in the phloem and immature xylem and is involved in regulating the formation of secondary cell walls [10]. Tomato *THyPRP* is specifically expressed in the abscission zone of the flower and regulates petal shedding by modulating the vigor of the deviated cells of the flower to respond to ethylene signals [11]. Citrus *CsPRP4* and its promoter are mainly expressed in leaves, and overexpression of this gene promotes starch accumulation in leaves [12]. PRPs were identified as cell wall proteins and they are involved in cell wall formation [5]. Recent studies showed that *PRPs* in many plants are related to biotic and abiotic stresses. For example, *FOCL1* in Arabidopsis thaliana encodes a *PRP* that is specifically expressed in guard cells. *FOCL1* mutants failed to form normal stomata; instead, stomata were covered by epidermal cells, which reduced plant transpiration and improved drought resistance [13]. *CcHyPRP* isolated from pigeon pea (*Cajanus cajan*) can be induced by various stresses. Overexpression of this gene in rice improved the resistance to rice Fusarium wilt, drought, salt, and heat stress [14,15]. Tomato *HyPRP1* negatively regulates plant tolerance to salt and oxidative stresses and participates in tomato sulfite metabolism [16]. When faced with low temperature challenges, transgenic plants with the expression of *PtrPRP* suppressed showed greater sensitivity to low temperature compared with that of wild plants, indicating that *PtrPRP* is a positive regulator in the cold response [17].

The aim of this study was to characterize the function of *PRPs* in apple under adverse environments. We conducted a genome-wide analysis of the apple genome to isolate all of the *PRP* genes and analyze their expression patterns. We also generated transgenic plants to assess the potential role of *MdPRP6* in the regulation of heat stress tolerance in plants. Our results will aid future functional analyses of *PRP* genes and the use of *PRP* transgenes to improve abiotic stress tolerance in apple.

## 2. Results

### 2.1. Genome-Wide Identification of PRP Family Members in Apple

After checking for the PRP domains using Pfam-downloaded HMM files, Hmmer software with default parameters and SMART online search platforms were used to examine the conserved PRP domain. Finally, nine genes were considered to be members of the PRP family in apple and renamed from *MdPRP1* to *MdPRP9*. All of the basic information for these 9 *MdPRPs* is provided in Table 1. The lengths of the CDS were between 378 bp (*MdPRP6*) and 1059 bp (*MdPRP2*). The predicted molecular weights (MW) and pI ranged from 12.85 kDa (MdPRP6) to 34.7 kDa (MdPRP3) and from 5.09 (*MdPRP6*) to 9.27 (*MdPRP3*), respectively. To further specify the *MdPRP* genes, we cloned all nucleotide sequences based on the predicted sequences. Details of the primers used for this assay are present in Supplemental Table S1.

### 2.2. Chromosomal Location of MdPRP Genes

**Figure 1:**
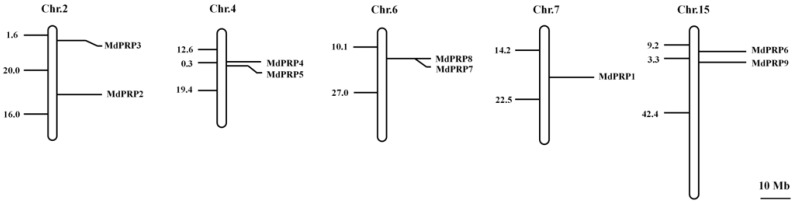
Chromosomal mapping of apple PRP family. Chromosome number is indicated at top of each chromosome. Numbers beside chromosomes suggest length of chromosome section pointed by line. Scale represents 10-Mb chromosomal distance.

The 9 *MdPRP* genes were displayed using MapInspect software according to their physical sites on the *Malus domestica* chromosomes. The 9 *MdPRP* family members were distributed on 5 of the 17 *M. domestica* chromosomes, including two (*MdPRP2* and *MdPRP3*) on Chr. 2, two (*MdPRP4* and *MdPRP5*) on Chr.4, two (*MdPRP7* and *MdPRP8*) on Chr. 6, one (*MdPRP1*) on Chr. 7, and two (*MdPRP6* and *MdPRP9*) on Chr. 15 (as illustrated in Figure 1).

### 2.3. Multiple Sequence Alignment and Analyses of the Evolution and Structure of MdPRP Genes 

**Figure 2:**
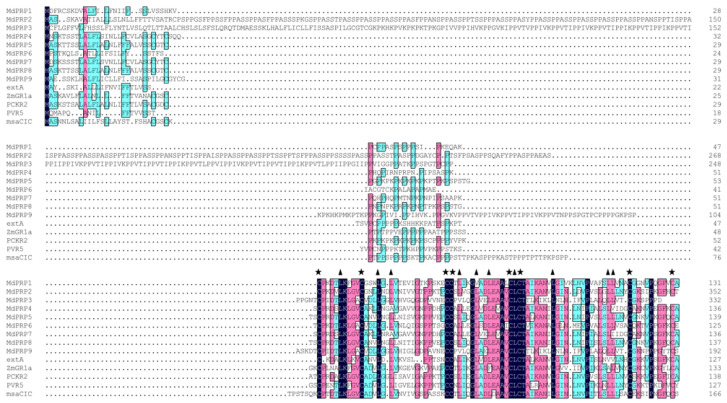
Comparison of deduced amino-acid sequence of MdPRP genes with that of other PRP proteins by DNAMAN software and numbered on the right. msaCIC (L22305), PCKR2 (X85206), extA (X67421), PVR5 (U34333), and ZmGR1a (AB018587) were from *Medicago sativa*, *Asparagus officinalis*, *Arabidopsis thaliana*, *Phaseolus vulgaris*, and *Zea mays*, respectively. Asterisks indicate conserved cysteine residue, and triangles indicate conserved Leucine residue in the cysteine-rich domain. Black, purple, and blue indicate that the similarity of each column of amino acids is 100%, 75%, and 50%, respectively.

To clarify the evolutionary relationships among *MdPRP* genes, we compared the full-length PRP protein sequences from *M. domestica*, *Arabidopsis thaliana*, *Medicago sativamsa*, *Asparagus officinalis*, *Phaseolus vulgaris*, and *Zea mays*. Multiple alignments revealed high similarity in their conserved domain: the first domain represented the putative signal peptide, a higher proportion of proline residues was located in the middle, and the last domain consisted of a long cysteine-rich hydrophobic domain (as illustrated in Figure 2). There are eight cysteines in the cysteine-rich hydrophobic domain. The spacing between the eight cysteines may be necessary to form disulfide bonds within or with other proteins [18,19]. Conservative spacing of L was also found in these PRP proteins and may be related to the formation of leucine zipper motifs [20]. These results indicate that PRP family protein sequences are highly conserved in plants.

**Figure 3:**
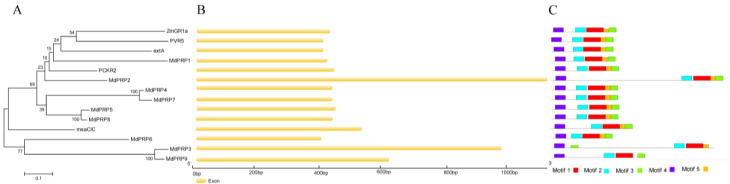
Comprehensive analysis of genes encoding arginine decarboxylase from genomes of *M. domestica*, *A. thaliana* (extA, X67421), *M. sativamsa* (msaCIC, L22305), *A. officinalis* (PCKR2, X85206), *P. vulgaris* (PVR5, U34333), and *Z. mays* (ZmGR1a, AB018587). (**A**) Phylogenetic relationship; phylogenetic tree was constructed using neighbor-joining method. (**B**) Gene structures of PRP; exon-intron analyses of PRP genes were carried out with GSDS. Lengths of exons of each PRP gene are exhibited proportionally, and (**C**) conserved motif of PRP proteins. Conserved motifs in PRP proteins were identified by MEME software. Each motif is indicated by a colored box numbered on the bottom. Length of motifs in each protein is presented proportionally.

To gain a better understanding of the evolutionary relationships among the MdPRP, we constructed an NJ phylogenetic tree of all MdPRP and five other plant protein sequences, as well as their gene structures and conserved motifs. Phylogenetic trees revealed that PRP proteins were highly conserved in the six plants (as illustrated in Figure 3A). The structure of intron-exon organization may cause differences in the coding region [21]. Thus, we explored the intron-exon organization of the 14 *PRP* genes and found none of the genes had introns (as illustrated in Figure 3B). In an attempt to obtain the divergence and function of the MdPRP proteins, we identified five conserved motifs using online MEME. Details of these five motifs are listed in Supplemental Table S2. All predicted conserved motifs were identified only once in each MdPRP protein and were found in all MdPRP proteins except MdPRP9 (as illustrated in Figure 3C).

### 2.4. Expression Patterns of MdPRP Genes in Different Tissues

**Figure 4:**
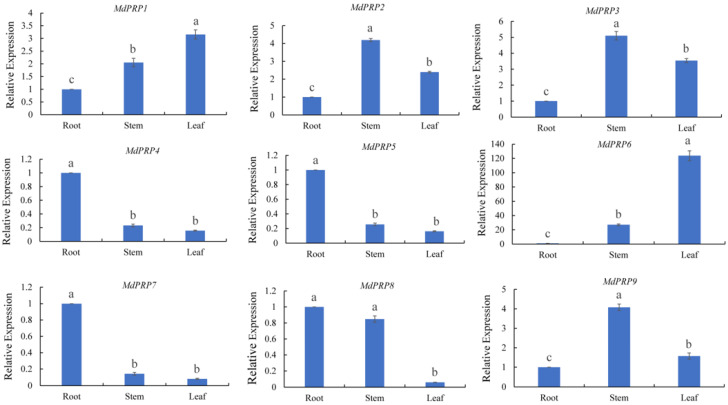
Relative expressions of *MdPRPs* in different tissues of *M. domestica cv. GaLa. MdMDH* primers were used as reference gene for each target gene. Transcript level at onset of each treatment was taken as 1, and those at other points were accordingly normalized and presented as relative fold changes. Different letters indicate significant differences among three tissues, according to one-way ANOVA and Tukey’s multiple range tests (*p* < 0.05). Each sample was analyzed in standard deviation of three biological replicates and three technical replicates in each biological sample.

The expression of *MdPRPs* was tested in the roots, stems, and leaves of apple. Most *MdPRP* genes were expressed in all tissues, except for four genes *(MdPRP4*, *-5*, *-7*, and *-8*), which showed low expression levels in all tissues and exhibited similar expression patterns (as illustrated in Figure 4). *MdPRP2*, *-3*, and *-9* were mainly expressed in the stem, and *MdPRP1* was most highly expressed in the leaves. *MdPRP6* was highly expressed in all tissues, especially in the leaves, and its expression was over 100-fold higher compared with that of the roots. The expression pattern of *MdPRP6* differed from that of the other *MdPRP* genes.

### 2.5. Expression Profiles of MdPRP Genes under Different Types of Stress

**Figure 5:**
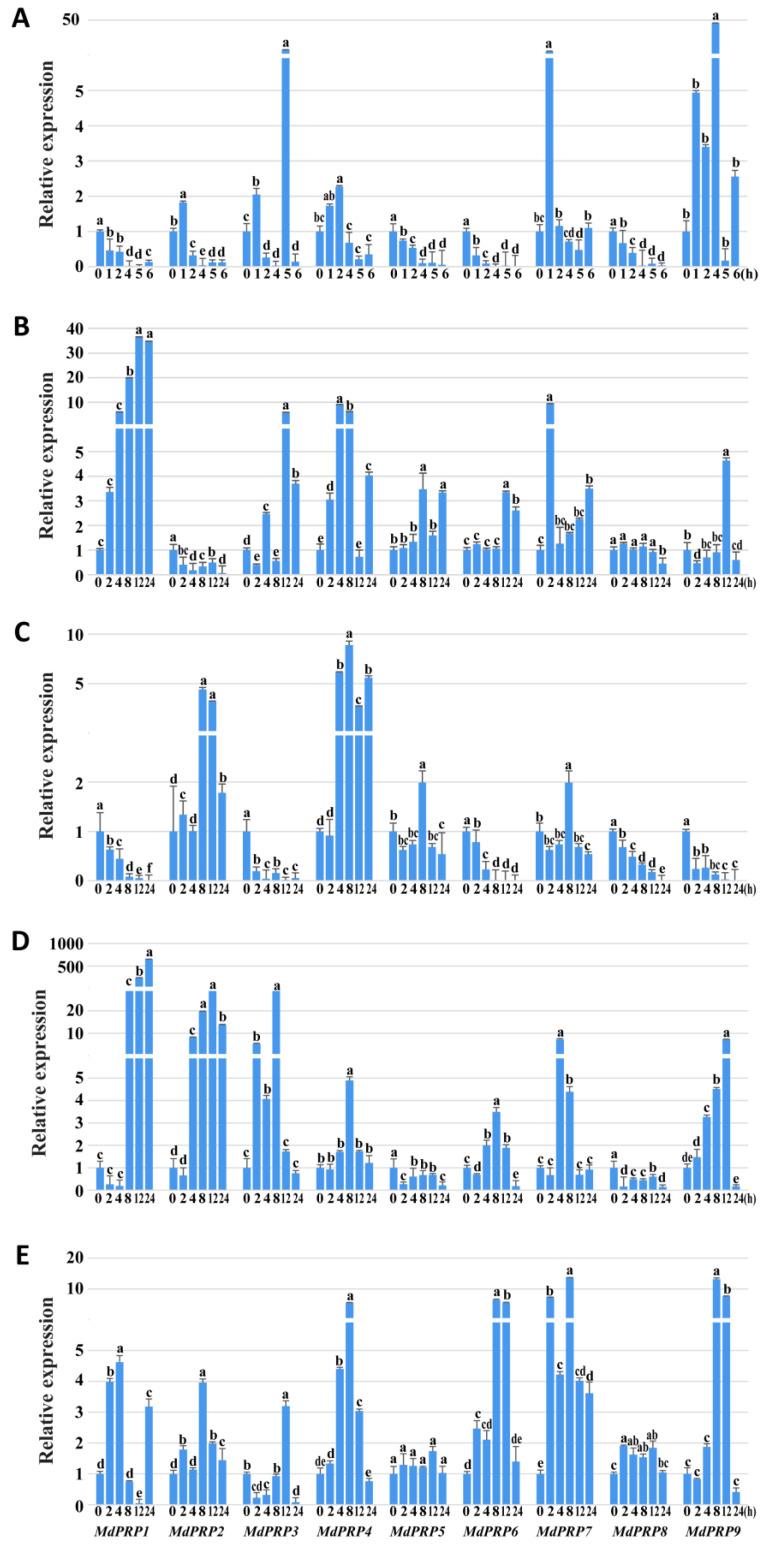
Relative expressions of *MdPRPs* in response to various stresses. (**A**) Heat treatment: treatment at 48 °C for 6 h, and the heat stress-treated leaves were collected at 0, 1, 2, 4, 5, 6 h; (**B**) cold stress: treatment at 4 °C for 24 h, and the cold-treated leaves were collected at 0, 2, 4, 8, 12, 24 h; for **(C**) drought stress, (**D**) salt stress, and (**E**) ABA stress, PEG6000 (10%; *w*/*v*), 150 mM NaCl, or 100 mM ABA were added, respectively, and the leaves were collected at 0, 2, 4, 8, 12, 24 h. *MdMDH* primers were used as the reference gene for each target gene. Transcript level at the 0 h in every treatment for each gene was taken as 1 when calculating relative expression levels, and those at other points were accordingly normalized and presented as relative fold changes. One-way ANOVA was used to compare statistical difference based on Tukey’s multiple range tests at significance levels of *p* < 0.05. Each sample was analyzed in standard deviation of three biological replicates and three technical replicates in each biological sample.

In plants, *PRPs* play important roles in response to various types of stress. qRT-PCR analysis was used to characterize the expression profiles of *MdPRP* genes under different types of abiotic stress. Expression patterns of *MdPRPs* varied under heat stress (as illustrated in Figure 5A). *MdPRP2, -4, -7*, and *-9* were induced by heat stress. The expression of *MdPRP9* was 50-fold higher at 8 h compared with its expression at 0 h. By contrast, the expression of *MdPRP1, -5, -6*, and *-8* was suppressed by heat stress. The expression of *MdPRP3* fluctuated under heat stress, increased during the first 2 h, and decreased in the next 8 h; it then suddenly peaked (over 5-fold) at 12 h and then decreased until 24 h.

The expression of *MdPRP1* was upregulated by cold stress and was higher compared with other genes during the same period (as illustrated in Figure 5B). *MdPRP2* was suppressed by cold stress, and *MdPRP8* was slightly affected by cold stress treatment; the expression level changed little during 24 h. The expression of the other *MdPRPs* also tended to be induced by cold, but their expression levels were lower compared with that of *MdPRP1*.

Drought stress treatment induced the expression of 4 *MdPRPs* and suppressed the expression of the five other *MdPRPs*. Specifically, the expression of *MdPRP2*, *-4*, *-5*, and *-7* was induced in response to drought. *MdPRP4* had the highest expression level during the stress period. The expression of *MdPRP1*, *-3*, *-6*, *-8*, and *-9* was downregulated by drought; their expression levels continuously declined since 0 h (as illustrated in Figure 5C).

The transcript levels of *MdPRP5* and *MdPRP8* changed slightly under saline conditions (as illustrated in Figure 5D). Following the initial suppression in the first 4 h, the expression of *MdPRP1* quickly increased until 24 h and increased as high as 600-fold compared with its expression at 0 h. The rest of the *MdPRPs*, including *MdPRP2*, *-3*, *-4*, *-6*, *-7*, and *-9*, showed similar expression profiles under drought; their expression levels first increased, peaked at different time points, and then decreased.

All of the *MdPRPs* tended to be induced by ABA treatment but to different degrees (as illustrated in Figure 5E); for example, the expression levels of *MdPRP5* and *MdPRP8* changed two-fold during the treatment. The second tier was composed of *MdPRP1, -2*, and *-3*; their expression levels were, at most, 3–5-fold higher compared with that of expression levels at 0 h. The greatest effect of ABA on the expression of *MdPRPs* was observed for *MdPRP4*, *-6*, *-7*, and *-9*, which rapidly responded to ABA and increased to nearly 10-fold compared with that of the expression level at 0 h.

### 2.6. Subcellular Localization of MdPRPs

**Figure 6:**
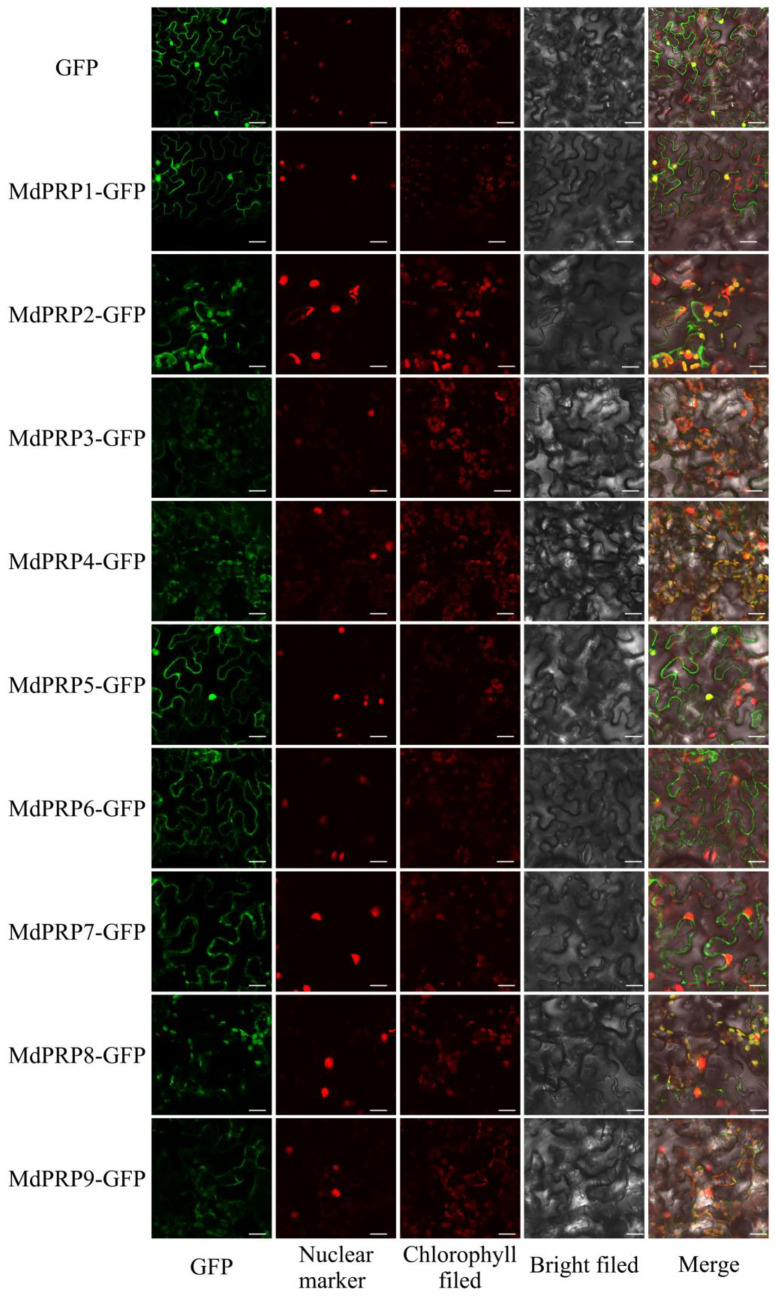
Subcellular localization analysis of MdPRPs. Fusion plasmid (35S MdPRP-GFP), with MdHB-7-mRFP driven by CaMV 35S promoter, were transiently cotransformed into tobacco epidermal cells. Fluorescence images were obtained using confocal microscopy (FV1000; Olympus, Tokyo, Japan). GFP image means Green fluorescent protein; Nuclear marker image means MdHB-7-mRFP (Red fluorescent protein); Chlorophyll field means Chloroplast autofluorescence; Merge means image GFP and image MdHB-7-mRFP and image chlorophyll merged with its bright-field photograph in same cell. Scale bar = 50 µm.

To investigate the subcellular localization of MdPRPs, the ORFs of MdPRPs were inserted into PGWB405 to produce 35S::GFP-MdPRPs and transiently coexpressed with MdHB-7-mRFP in *N. benthamiana* (as illustrated in Figure 6). Empty PGWB405-GFP was used as a negative control. Studies reported that MdHB-7 was located in the nucleus [22]; we used it as a nuclear maker. MdPRP1 and MdPRP5 were mainly anchored to the nucleus and cell membrane. MdPRP2, -3, -4, and -9 were mainly distributed on the cell membrane and chloroplast. The fluorescence of GFP-MdPRP6 and GFP-MdPRP7 fusion proteins was detected on cell membranes; in addition, the weak fluorescence was detected on chloroplasts in cells expressing GFP-MdPRP7 fusion protein. MdPRP8 was localized to the chloroplast. Most of the MdPRPs were observed in the cytoplasm, indicating that they are secretory proteins in apple.

### 2.7. Overexpression of MdPRP6 Improves Basal Thermotolerance in Transgenic Tobacco

**Figure 7:**
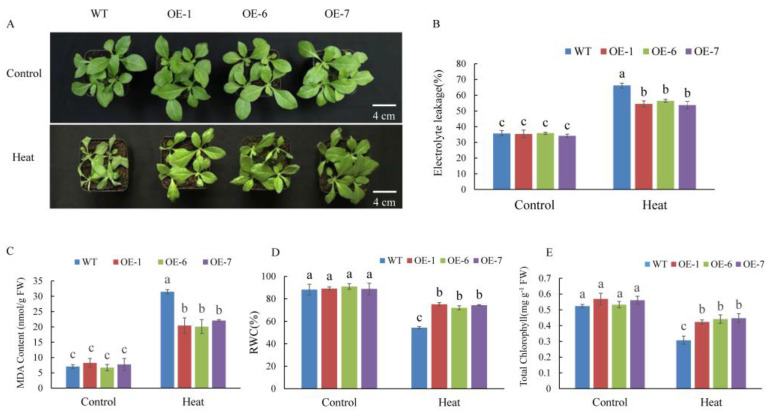
Overexpression of *MdPRP6* confers enhanced heat tolerance to transgenic tobacco plants. (**A**) Growth phenotypes of WT and transgenic lines (Line1, Line6, and Line7) under a normal temperature and after 48 °C for 6 h. (**B**) Electrolyte leakage, (**C**) malondialdehyde (MDA) concentration, (**D**) relative water content (RWC), and (**E**) chlorophyll concentration in WT and transgenic apples treated with or without high temperature. Scale bar = 4 cm. Data are means of three replicates with SD. Different letters indicate significant differences between WT and *MdPRP6* transgenic plants with or without heat stress (48 °C, 6 h), according to one-way ANOVA and Tukey’s multiple range tests (*p* < 0.05).

**Figure 8:**
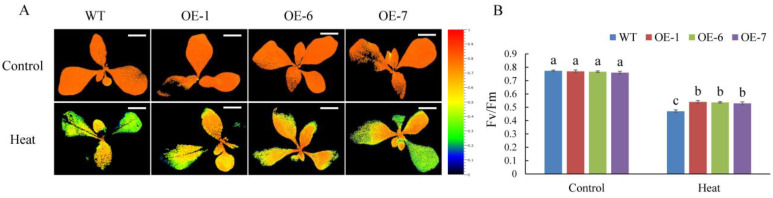
Overexpression of *MdPRP6* enhanced the Fv/Fm after 6 h under control and heat conditions. (**A**) Fv/Fm image obtained from chlorophyll fluorescence imaging and (**B**) Fv/Fm. Scale bar = 3 cm. Data are means of three replicates with SD. Different letters indicate significant differences between WT and *MdPRP6* transgenic plants with or without heat stress (48 °C, 6 h), according to one-way ANOVA and Tukey’s multiple range tests (*p* < 0.05).

Among all of the MdPRPs, MdPRP6 showed the strongest fluorescence on the cell membrane, indicating that it may enhance the function of cell membrane and the response to environmental stimuli. We thus generated transgenic tobacco plants overexpressing *MdPRP6* via the *Agrobacterium*-mediated transformation method. Transgenic lines were confirmed using PCR with genomic DNA as template, and the expression level of *MdPRP6* in different lines was quantitatively detected with qRT-PCR (as illustrated in Supplemental Figure S1). Next, three transgenic lines (OE-1, OE-6, and OE-7) with much higher *MdPRP6* expression levels were selected for heat stress treatment. Before high temperature treatment, both WT and *MdPRP6*-OE plants grew vigorously, and the leaves were stretched and bright green. After 6 h of 48 °C treatment, the WT suffered many more injuries, including curled and severely shriveled leaves, but the OE lines showed less damage, with only some leaves curled and slightly wilted (as illustrated in Figure 7). Consistent with the different phenotypes of WT and OE plants, significantly lower EL levels, reduced MDA accumulation, and higher chlorophyll concentrations and RWC levels were detected in OE plants compared with WT plants. As heat stress can damage PSII and affect photosynthetic electron transfer, we measured the maximum photochemical efficiency of PSII photochemistry (F_v_/F_m_). After heat stress, the images of chlorophyll fluorescence in OE plants displayed a wider distribution of orange. The qualitative F_v_/F_m_ decreased by 53% in WT plants and by ∼47% in the three OE lines (as illustrated in Figure 8). These results indicated that *MdPRP6* expression in transgenic tobacco conferred enhanced stress tolerance to high temperature compared with WT plants.

### 2.8. Overexpression of MdPRP6 in Tobacco Enhanced Antioxidase Activity 

**Figure 9:**
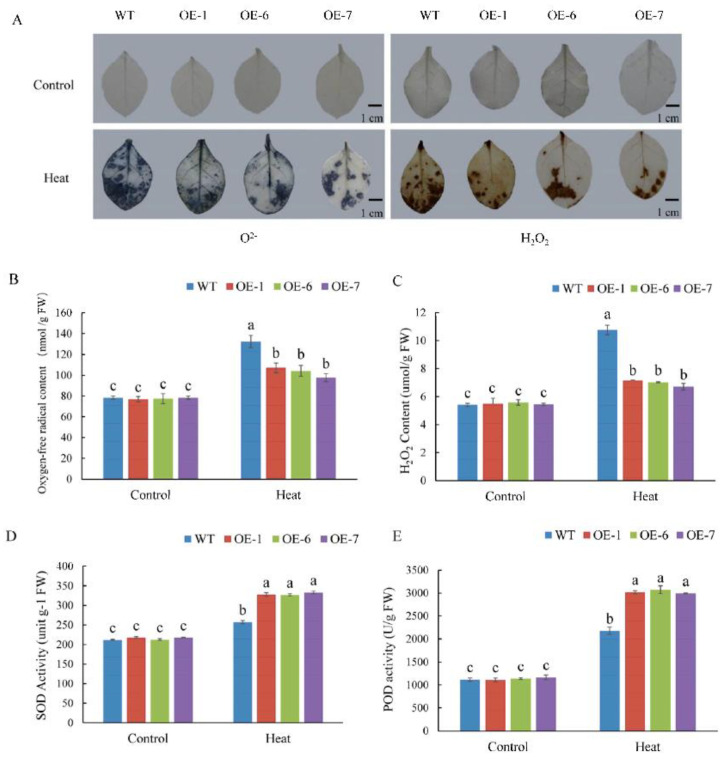
Overexpression of *MdPRP6* alleviated oxidative damage in tobacco under heat stress. (**A**) DAB and NBT staining. (**B**) O^2−^ concentrations; (**C**) H_2_O_2_ concentrations; (**D**) Activities of superoxide dismutase (SOD), and (**E**) activities of peroxidase (POD). Scale bar = 1 cm. Data are means of three replicates with SD. Different letters indicate significant differences between WT and *MdPRP6* transgenic plants with or without heat stress (48 °C, 6 h), according to one-way ANOVA and Tukey’s multiple range tests (*p* < 0.05).

Under high temperature stress, ROS accumulate in plants, which creates oxidative stress in plant cells. Here, DAB and NBT were used to characterize the accumulation of H_2_O_2_ and O^2–^, respectively. The results of histochemical staining showed that after 6 h of high temperature treatment, ROS accumulated in the leaves of WT and OE lines, but the degree of accumulation was significantly lower in the latter, as indicated by the smaller distribution of lighter blue and brown in leaves, respectively (as illustrated in Figure 9A). Quantitative measurements also showed that the accumulation of O^2–^ and H_2_O_2_ was significantly higher in WT plants than in OE lines (as illustrated in Figure 9B,C). By contrast, the detected peroxidase (POD) and superoxide dismutase (SOD) levels were lower in WT plants than in OE lines (as illustrated in Figure 9D,E). The above results indicated that *MdPRP6* overexpression in tobacco reduced the oxidative damage to plant cells, thereby enhancing tolerance to heat stress.

## 3. Discussion

Following the completion of the sequencing of the apple genome [23,24], many gene families were identified and characterized at the whole-genome level, including SAUR [25], Aux/IAA [26], MYB [27], bZIP [28], WRKY [29], and bHLHs [30]. In this study, genome-wide analyses resulted in the identification of nine *MdPRP* genes in the apple genome, which were named *MdPRP1-9*. Expression analysis permitted the identification of genes that play an important role in the response to various stresses. Our results indicated that the 9 *MdPRPs* were randomly distributed in the 5–17 chromosomes (as illustrated in Figure 1). Multiple alignment revealed high similarity in the conserved domain among *Arabidopsis, M. sativamsa, A. officinalis, P. vulgaris*, and *Z. mays*, which consisted of a signal peptide at the N-terminus and a hydrophobic cysteine-rich region in the C-terminus of each (as illustrated in Figure 2), suggesting that PRP proteins may be secreted to the extracellular matrix through the N-terminus signal peptide. In addition, the distance between leucine residues in the cysteine-rich region was relatively conservative, indicating that they may participate in the formation of leucine zipper elements as in other plants [31]. An unrooted phylogenetic tree showed that the homologous gene pairs with high sequence identities exhibited close evolutionary relationships. The results of the analysis of intron-exon structure organization were similar to those of nine *MdPRP* genes in other plants (as illustrated in Figure 3).

Previous studies noted the tissue-specific expression of *PRP* in many plants [9]. Consistent with our work, *MdPRPs* were expressed ubiquitously in different tissues and organs. The qRT-PCR analysis demonstrated different expression patterns of 9 *MdPRP* genes in the root, stem, and leaves of apple. For example, *MdPRP2*, *-3*, and *-9* were mainly expressed in the stem, and *MdPRP1* and *MdPRP6* were mainly expressed in the leaves (as illustrated in Figure 4). The expression of *MdPRPs* in different tissues in apple suggests that *MdPRPs* may function in multiple physiological processes. In this study, we also noticed that *MdPRPs* responded to heat, cold, drought, salt, and ABA (as illustrated in Figure 5). These results indicated that *MdPRPs* were sensitive to various stresses, but the response differed among various environmental stimuli. This phenomenon was also observed for other *PRP* genes. For example, low temperatures induced the expression of *BnPRP* in rape, and high temperature, drought, and injury did not affect its expression [32]. Among the 18 *PRP* genes in *Arabidopsis*, nematode infection upregulated the expression of *PRP4*, *PRP11*, and *PRP16*, and *P. syringae* infection induced the expression of *PRP9* and *PRP10* [33]. *JsPRP1* overexpression was shown to confer strong resistance to drought stress, CdCl_2_ stress, and *C. gloeosporioides* infection [34]. These findings suggested that plant *PRP*s were differentially regulated under abiotic stresses and different members of the *PRP* family might play specific roles in mediating abiotic stress. PRPs were reported to locate on the cell wall and plasma membrane and possess important roles for forming plant cell walls and responding to environmental stress [35,36,37]. In *Arabidopsis*, HyPRP was identified to primarily be located on the cell wall, but it also interacts with the cell membrane [38]. The subcellular location analysis revealed that the GhHyPRP3 protein was mainly an anchor to the plasma membrane [39]. Citrus CsPRP4 protein was localized in the plasma membrane [12]. Subcellular analysis showed the localization of JsPRP1 in the onion epidermal cells [33]. Similar to the informed studies, most of the MdPRP proteins can be detected in the cell membrane, and several members were also located in the nucleus or chloroplasts (as illustrated in Figure 6). This indicated that MdPRP proteins function in various parts of the cell.

Under high temperature stress, plants experience several types of damage, including protein denaturation and misfolding, damage to the cell structure and photosynthetic system, and the large accumulation of ROS [40,41,42,43]. The transcription level of *MdPRP6* decreased under high temperature stress, indicating that it may be involved in the response to high temperature stress. To analyze the specific role of *MdPRP6* in response to high temperature, both WT and heterologously overexpressed *MdPRP6 N. nudicaulia* were subjected to high temperature treatment. Under high temperature treatment, transgenic plants experienced less damage and were more resistant to high temperature compared with that of WT plants (as illustrated in Figure 7A). This indicates that *MdPRP6* participated in the defense response of plants under high temperature resistance. The transcription level of *MdPRP6* was downregulated in response to heat stress, which may reflect posttranscriptional or posttranslational modifications. This is a subject that we plan to examine further.

Heat stress seriously affects the growth and development of plants and can damage the plant membranes. EL indicates the permeability of plant membranes, and the content of MDA can reflect the degree of membrane lipid peroxidation, both of which are often used as indicators of plant membrane integrity and damage [44,45,46]. Under high temperature stress, the EL and MDA content were significantly lower in *MdPRP6*-overexpressing plants than in WT plants, indicating that the transgenic plants were less damaged under high temperature stress (as illustrated in Figure 7B,C). Therefore, after the heterologous overexpression of *MdPRP6* in tobacco, the efficiency of removing damaged oxidized proteins on the membrane was higher under high temperature stress, thereby alleviating damage to the membrane system of transgenic plants. High temperature stress also damages plant photosystem II, and thus affects F_v_/F_m_, photosynthetic electron transport and ATP synthesis, resulting in a decrease in the quantum yield of plant PSI and PSII systems [47,48]. The decrease in photosynthetic ability caused by heat stress directly affects the photosynthetic apparatus by reducing F_v_/F_m_ [49]. Similar results were also obtained in this study: OE lines had higher F_v_/F_m_ values than WT lines under heat stress conditions (as illustrated in Figure 8).

Under high temperature stress, a large amount of ROS species accumulated in plants, which causes oxidative stress; O^2−^ and H_2_O_2_ are important ROS species [50]. The accumulation of O^2−^ and H_2_O_2_ was lower in *MdPRP6*-overexpressing plants than in WT plants (as illustrated in Figure 9A–C), and the activity of SOD and POD was significantly higher in transgenic plants than in WT plants under high temperature stress (as illustrated in Figure 9D,E). Many studies have shown that the ROS regulation system of plants is important for responding to high temperature stress. For example, *Arabidopsis thaliana* ROS metabolic mutants lacking an antioxidant system were highly resistant to high temperature [51]. The heterologous expression of wheat F-box protein in tobacco enhanced the activity of ROS scavenging enzymes in transgenic plants, reduced ROS accumulation, and improved the resistance of transgenic plants to high temperature stress [52]. The higher temperature resistance of transgenic plants may stem from changes in the metabolism of active oxygen. Under high temperature stress, overexpression of *MdPRP6* could efficiently remove active oxygen, thereby alleviating the damage caused by the accumulation of active oxygen.

## 4. Materials and Methods

### 4.1. Identification of Putative PRP Family Members in Malus Domestica

The identification of PRP proteins in the apple genome was achieved by HMMER software (version 3.1b2; http://hmmer.org/, UC Santa Cruz) using the hidden Markov model (HMM) to computationally compare all proteins in the genome. Briefly, we downloaded the HMM file of the PRP domain from the Pfam (http://pfam.xfam.org/) (accessed on October 2019, latest update on March 2021) [53] as a query to search the protein sequences via HMMER software with the default E-value. The protein sequences of PRP from HMMER software was validated using the SMART (http://smart.embl-heidelberg.de/) (accessed on October 2019, latest update in 2020) [54]. The redundant sequences were manually removed.

### 4.2. Bioinformatics Analysis of PRP Genes

Locations of the *MdPRP* gene on the apple different chromosomes was shown using MapDraw. The lengths, masses, and isoelectric point (pI) values for MdPRP were tested with Editseq software. The exon-intron structures of each *MdPRP* were obtained from the online Gene Structure Display Server (GSDS: http://gsds.cbi.pku.edu.cn) (accessed on October 2019, latest update on 10 December 2014); We identified conserved motifs in MdPRP protein using online MEME (http://meme-suite.org/) (accessed on October 2019, latest update on 21 February 2021). Sequence alignment analysis of the PRP was performed using MUSCLE in MEGA 6.06 software with default parameters; the evolutionary tree of PRP was constructed by MEGA 6.06 software according to the neighbor-joining method, and the test parameter Bootstrap was repeated 1000 times.

### 4.3. Plant Materials and Treatments

The Gala seedlings were grown in a chamber with a 14 h light/10 h dark photoperiod and 40–60% relative humidity at 23 °C. After 30 days, seedlings with the same growth state were selected and transferred to hydroponic culture (Hoagland nutrient solution). Stress treatments were applied when the plants grew new roots. For drought, salt, and ABA stress treatment, PEG6000 (10%; *w*/*v*), 150 mM NaCl, and 100 mM ABA were added, respectively. To induce low temperature stress, more plants were transferred to an incubator set at 4 °C for 24 h. To induce high temperature stress, the plants were transferred to an incubator set at 48 °C for 6 h. The heat stress-treated leaves were collected at 0, 1, 2, 4, 5, 6 h and the others were collected at 0, 2, 4, 8, 12, 24 h. All of the samples were stored at −80 °C.

### 4.4. RNA Extraction and qRT-PCR 

The total RNA of leaves was extracted using the Wolact plant RNA isolation kit (Wolact, Hongkong, China). The quality and concentration of the extracted RNA were measured by a BioPhotometer (D30, Eppendorf, Germany). cDNA synthesis was carried out using a Reverse Transcription Kit (Thermo Scientific, Waltham, MA, USA). A LightCycler 96 quantitative instrument (Roche, Switzerland) was used for quantitative real time-PCR (qRT-PCR). For the different tissues expression, and expression profiles under stress conditions of *MdPRPs*, *MdMDH* was used as the reference gene to calculate relative expression of target genes [55]. The expression levels of *MdPRP6* in overexpression tobacco lines was detected by qRT-PCR using *Ubiquitin* as the internal control [56]. the specific primers were designed based on the *MdPRP* gene sequences. All primers were shown in Supplemental Table S1. Three biological replicates were carried out, and each biological replicate contained three technical replicates.

### 4.5. Genetic Transformation of MdPRP 

The CDS of the MdPRP6 gene were constructed to the pGWB415 vector to form a MdPRP-HA fusion protein expression vector, which was introduced into *Agrobacterium tumefaciens* strain EHA105 by the freeze and thaw method. Transformation of *Nicotiana nudicaulia* was performed using the leaf disc transformation method according to Gong et al. (2015) [57]. Transgenic lines were selected by 50 mg L^−1^ spectinomycin and spectinomycin-resistant plants were determined by DNA level. The expression levels of MdPRP6 in overexpression lines were confirmed by qRT–PCR. T3 transgenic plants were used for subsequent experiments. To confirm the subcellular localization of MdPRPs, the full length open reading frames (ORFs) of 9 MdPRPs were cloned into the PGWB405 vector to generate MdPRPs-GFP, and the negative control used was PGWB405 empty vector. We constructed MdHB-7 on the PGWB455 vector to generate MdHB-7-mRFP as a nuclear maker. The freeze and thaw method was used to transform all genes into *Agrobacterium* strain EHA105. Transformed *Agrobacterium* PGWB405-GFP and 9 MdPRPs-GFP with MdHB-7-mRFP were coinfiltrated into the leaves of *N. benthamiana* following a previous study [58]. Samples were analyzed by a confocal microscope (FV1000; Olympus, Tokyo, Japan). All primers are listed in Supplemental Table S1

### 4.6. Heat Stress Treatment of Transgenic Tobacco

Four-week-old seedlings of *N. nudicaulia* were used for assays of heat stress tolerance. Seedlings of *N. nudicaulia* were cultured in a growth chamber under a 14 h light/10 h dark photoperiod at 23 °C. For the heat stress treatment, the temperature of the light incubator was adjusted to 48 °C for 6 h. Electrolyte leakage (EL) of leaves was measured according to Sun et al. (2018) [59]. According to previous method, tobacco leaves were excised and scored according to their fresh weight, rehydrated and dry weights, and RWC = (fresh weight − dry weight)/(rehydrated weight−dry weight)”, and this was used to calculate RWC of leaves [60]. Chlorophyll content was determined by UV-2250 spectrophotometer (Shimadzu, Kyoto, Japan) after leaf chlorophyll was extracted with 80% acetone, as described by Liu et al. (2021) [61]. The activities of SOD, POD, and the MDA, H_2_O_2_, and O^2−^ content, were detected with their corresponding detection kits (Suzhou Comin Biotechnology Co., Ltd., Suzhou, China). Each experiment was repeated three times.

### 4.7. Statistical Analysis

SPSS software (IBM, Chicago, IL, USA) was used for all statistical analysis. One-way ANOVA was used to complete the results. Differences were detected statistically significant at *p* < 0.05.

## 5. Conclusions

Our paper presented a genomics survey of the *PRP* genes in apple and identified nine *MdPRPs* in the apple genome. They were expressed in various tissues and under different types of abiotic stress, such as heat, cold, drought, salt, and ABA treatment. In addition, the nine MdPRPs were distributed in different areas of the cell, mainly in cytoplasm, indicating that they were secretory proteins with signal peptides. Additional analyses demonstrated that *MdPRP6* positively regulated heat stress tolerance in transgenic tobacco plants. The OE plants showed less damage, higher photosynthetic capacity, and higher activity of antioxidative enzymes (SOD and POD), suggesting that PRP proteins in apple are involved in the response to abiotic stress. These findings will aid future studies aimed at characterizing the functional mechanisms by which PRP proteins in apple facilitate responses to adverse environments.

## Figures and Tables

**Table 1:** List of identified *PRP* genes in *Malus domestica* with their detailed information.

Name	Locus ID	CDs Lengths (bp)	Size (aa)	Mass (KDa)	pI	Chromosome	Location
MdPRP1	MD07G1116600	396	396	131	8.18	chr7: 14184587	14184982
MdPRP2	MD02G1209700	1059	1059	35	7.78	chr2: 21580073	21581173
MdPRP3	MD02G1023200	999	999	333	9.27	chr2: 1622540	1624717
MdPRP4	MD04G1086500	411	411	137	7.97	chr4: 12600404	12600814
MdPRP5	MD04G1087300	420	420	140	9.05	chr4: 12905035	12905603
MdPRP6	MD15G1126700	378	378	126	5.09	chr15: 9208073	9208450
MdPRP7	MD06G1062200	411	411	137	7.97	chr6: 10139920	10140330
MdPRP8	MD06G1062000	414	414	138	8.64	chr6: 10095205	10096031
MdPRP9	MD15G1166100	582	582	194	8.69	chr15: 12485068	12486646

## Data Availability

Not applicable.

## Supplementary Materials

The following are available online at https://www.mdpi.com/article/10.3390/ijms22115942/s1.
