# Supplementary material for: Genome-Wide Identification of PRP Genes in Apple Genome and the Role of MdPRP6 in Response to Heat Stress
Source: Int J Mol Sci. 2021 May 31;22(11):5942. doi: 10.3390/ijms22115942 (PMC8198058; doi:10.3390/ijms22115942)

Supplemental Table 1. Application of primers and sequences

**Primer Name**	**Purpose/Vector**	**Sequence (5'-3')**
MdPRP1		F: ATGGACTTTAGGTGTTC
		R: TTAAGCACAGACAAAGC
MdPRP2		F: ATGGCTTCTTCTAAGGCAGTTG
		R: TTAGGTGCAATGGAAGCCTT
MdPRP3		F: ATGTGCTTCCTAGGGTTTT
		R: TCAATCAGGAGGAGGACT
MdPRP4		F: ATGAATTCCAAAACATCTTC
		R: TTATGCACATTTGAAGCCCT
MdPRP5	**Clone/pMD19T-simple**	F: ATGGCATCCAAAACGAC
		R: TTAGGCACACTGGAAACC
MdPRP6		F: ATGCCTTCTACCAAGCAGCTCTC
		R: TCATTCACATTTGAAGCCAGGAGG
MdPRP7		F: ATGGATTCCAAAAGTTCTTC
		R: TTATGCACATTTGAAGCC
MdPRP8		F: ATGGCATCCAAAACAACTTC
		R: TTAGGCACATTGGAAACC
MdPRP9		F: ATGGCGGAATCCAGCAA
		R: CTAGAGAGAGCAAGTGTAACC
MdPRP1		F: GGGGACAAGTTTGTACAAAAAAGCAGGCTTCATGGACTTTAGGTGTTC
		R: GGGGACCACTTTGTACAAGAAAGCTGGGTTAGCACAGACAAAGC
MdPRP2		F: GGGGACAAGTTTGTACAAAAAAGCAGGCTTCATGGCTTCTTCTAAGGCAG
		R: GGGGACCACTTTGTACAAGAAAGCTGGGTT GGTGCAATGGAAGCCTT
MdPRP3		F: GGGGACAAGTTTGTACAAAAAAGCAGGCTTCATGTGCTTCCTAGGGTTTT
		R: GGGGACCACTTTGTACAAGAAAGCTGGGTTATCAGGAGGAGGACT
MdPRP4		F: GGGGACAAGTTTGTACAAAAAAGCAGGCTTCATGAATTCCAAAACATCTT
		R: GGGGACCACTTTGTACAAGAAAGCTGGGTTTGCACATTTGAAGCCCT
MdPRP5	**PGWB405/415**	F: GGGGACAAGTTTGTACAAAAAAGCAGGCTTCATGGCATCCAAAACGAC
		R: GGGGACCACTTTGTACAAGAAAGCTGGGTTGGCACACTGGAAACC
MdPRP6		F: GGGGACAAGTTTGTACAAAAAAGCAGGCTTCATGCCTTCTACCAAGCAG
		R: GGGGACCACTTTGTACAAGAAAGCTGGGTTTTCACATTTGAAGCCAGGA
MdPRP7		F: GGGGACAAGTTTGTACAAAAAAGCAGGCTTCATGGATTCCAAAAGTTCTT
		R: GGGGACCACTTTGTACAAGAAAGCTGGGTTTGCACATTTGAAGCC
MdPRP8		F: GGGGACAAGTTTGTACAAAAAAGCAGGCTTCATGGCATCCAAAACAACT
		R: GGGGACCACTTTGTACAAGAAAGCTGGGTTGGCACATTGGAAACC
MdPRP9		F: GGGGACAAGTTTGTACAAAAAAGCAGGCTTCATGGCGGAATCCAGCAA
		R: GGGGACCACTTTGTACAAGAAAGCTGGGTTGAGAGAGCAAGTGTAACC
MdHB-7	**PGWB455**	F: GGGGACAAGTTTGTACAAAAAAGCAGGCTTCATGCTCGATAAAATCGAAT
		R: GGGGACCACTTTGTACAAGAAAGCTGGGTTGAGACCAGAAGTCCCACC
35S		GCTCCTACAAATGCCATCA

MdPRP1		F: CCTTCCCCGCCTCCCTCTATTC
		R: CCCTAACCAACTCCCACAAACACC
MdPRP2		F: GTAGCCTCGTTGGTGGACTTGC
		R: GGAAGCCTTTCGGGATAGACTTG
MdPRP3		F: GCCAAAGCACAAGCCTGTTAAACC
		R: TTACGATGGGAGGGACTGTGACTG
MdPRP4	**qRT-PCR**	F: CCCACCAGATCACCCTTGTT
		R: CTCGGGGACATTCTTCGCAC
MdPRP5		F: GCCCTTGTCAGTTCTTGTGG
		R: AGCAAGGTTCTACGGGAGGT
MdPRP6		F: CGCTAGGGCTCAACATGGAA
		R: TTTGAAGCCAGGAGGGACTG
MdPRP7		F: CACCCTTGTTGTGCTTTGCT
		R: ATTTGAAGCCCGAAGGGACAT
MdPRP8		F: CAAAGCCAAATCCGACGCCA
		R: CAGCGAGCCCTTGGATGAGA
MdPRP9		F: GCAAGCCAAAGCACAAGCCTATG
		R: GTGGTTTGACAATGGGAGGGACTG
MDH		F: ATTCAAGTATGCCTGGGTGC
		R: CAGTCAGCCTGTGATGTTCC
Ubiquitin		F: GGTGTTTCCAGTGGCGGACG
		R: TCCTCCCCTCAGCTACGGGGTAT

Supplemental Table 2. Conserved motifs in the MdPRP proteins

Motif	Width	E-value	Logo
1	35	16.7e-216	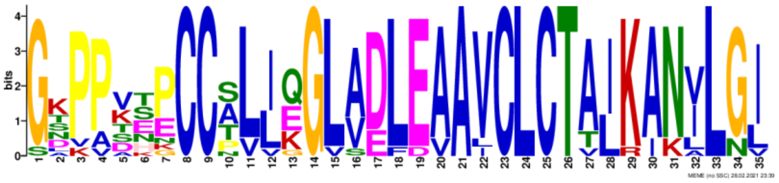
2	21	2.9e-098	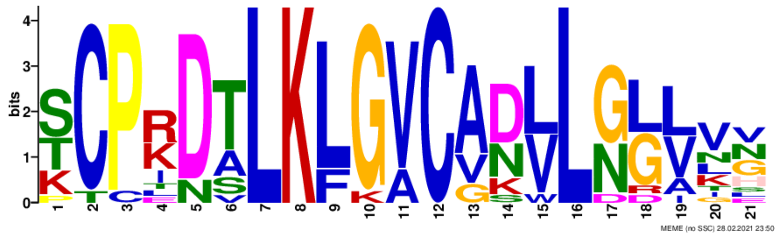

3	15	6.7e-051	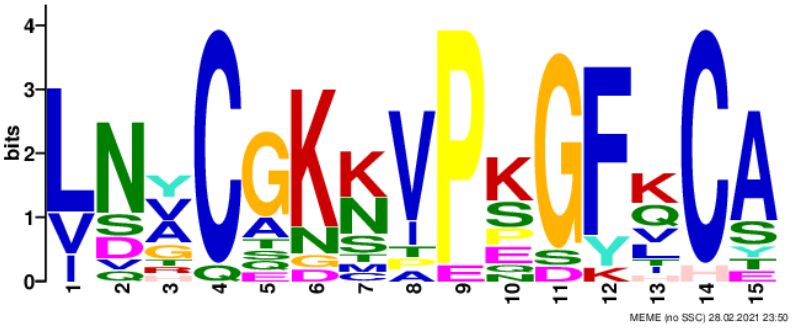
4	21	4.4e-045	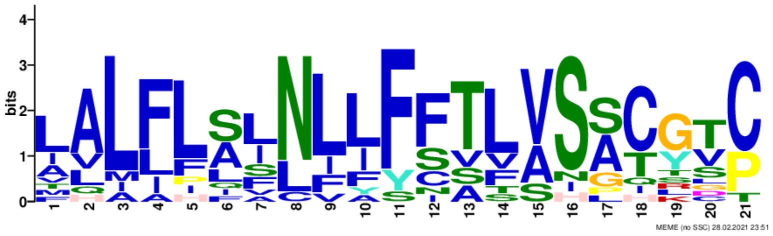
5	11	4.6e-022	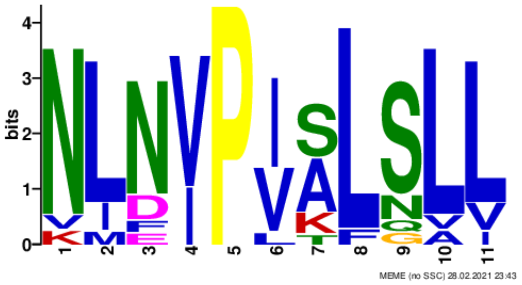

Supplemental Figure. 1. PCR identification and relative expression analysis of *MdPRP* in transgenic *tobacco*

A

B

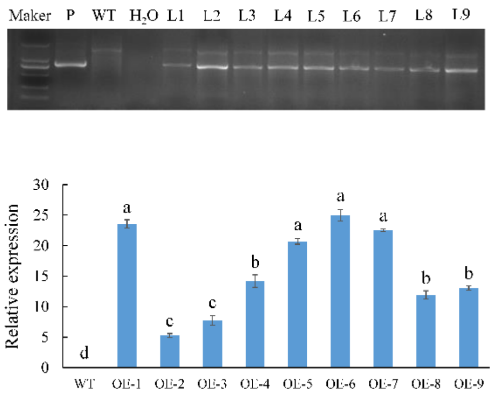

Supplement: Supplementary file 1 [file ijms-22-05942-s001.zip › ijms-1218505-supplementary.pdf]
